# Supplementary figures and images for: Retinoic Acid Induced Protein 14 (Rai14) is dispensable for mouse spermatogenesis
Source: PeerJ. 2021 Feb 19;9:e10847. doi: 10.7717/peerj.10847 (PMC7899019; doi:10.7717/peerj.10847)

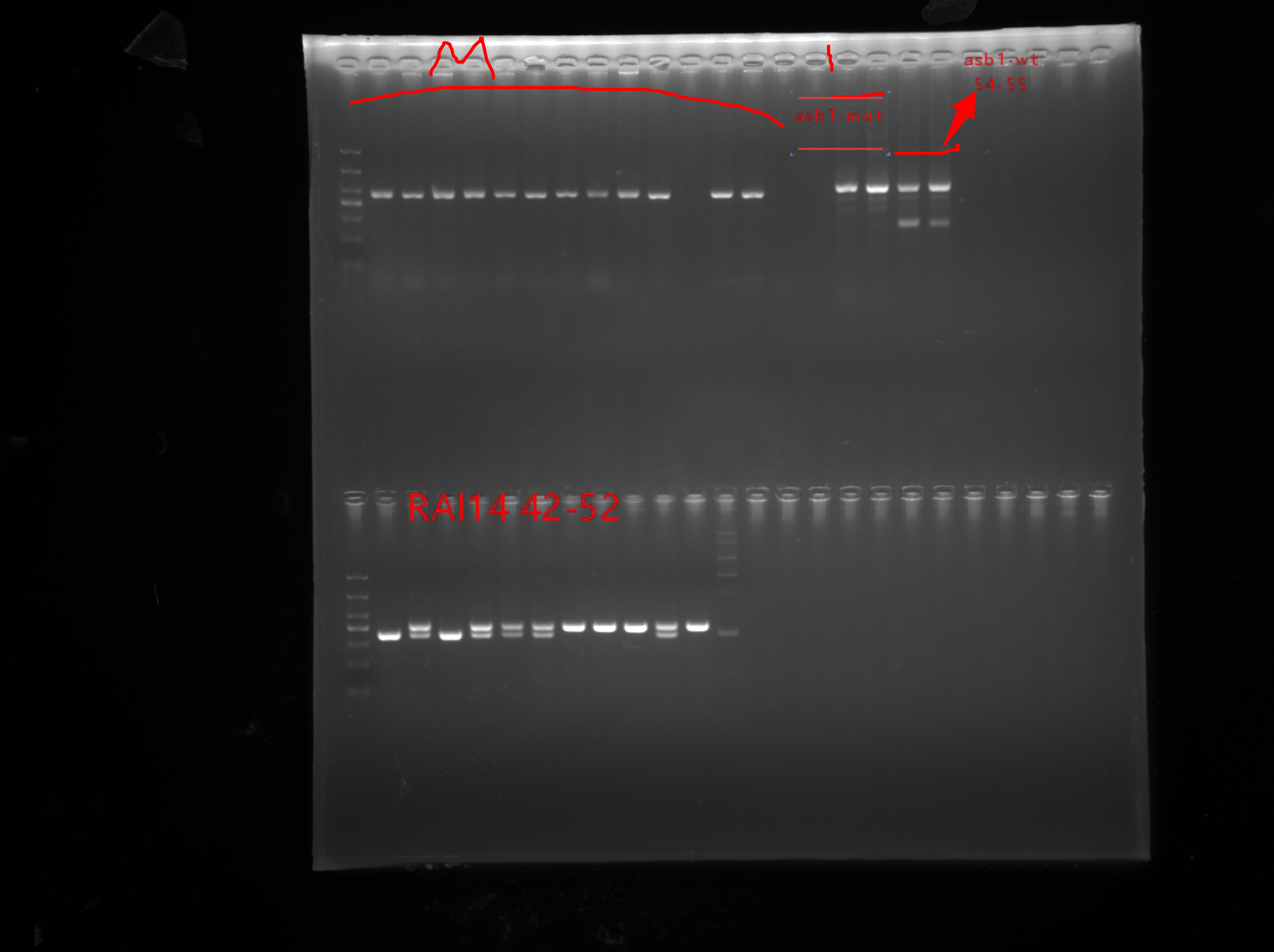

Supplement: Supplemental Information 6 — Full-length uncropped gels and blots. [file peerj-09-10847-s006.zip › Gels of Fig.1B.jpg]

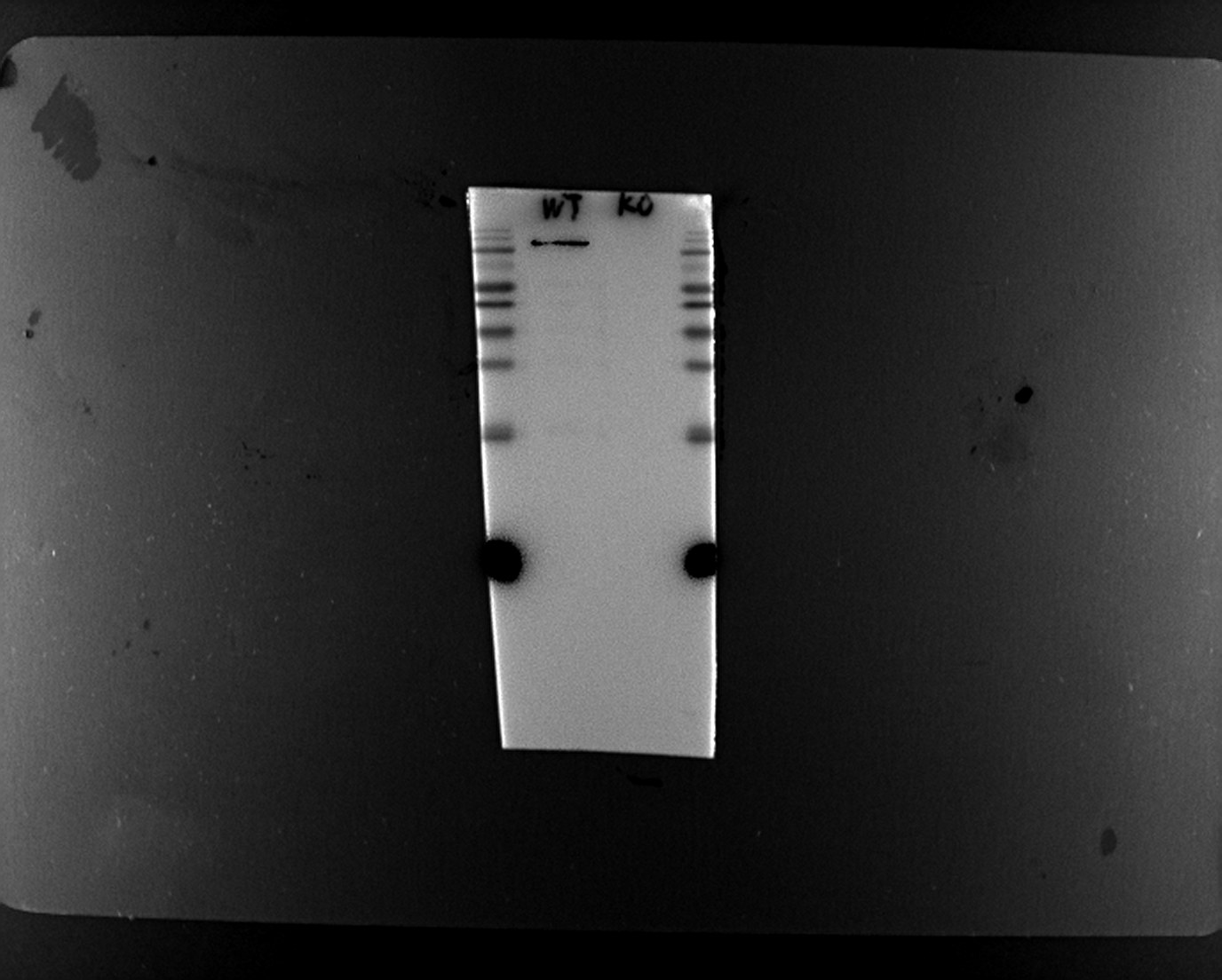

Supplement: Supplemental Information 6 — Full-length uncropped gels and blots. [file peerj-09-10847-s006.zip › Blots of Fig. 1C.tif]
